# Comparative Efficacy, Safety, Tolerability, and Effectiveness of Antipsychotics in The Treatment of Dementia-Related Psychosis (DRP): A Systematic Literature Review

**DOI:** 10.14283/jpad.2021.48

**Published:** 2021-08-06

**Authors:** I. Yunusa, N. Rashid, V. Abler, Krithika Rajagopalan

**Affiliations:** 1 Center for Outcomes Research & Evaluation, University of South Carolina College of Pharmacy, Columbia, South Carolina, USA; 2 Acadia Pharmaceuticals, Inc., San Diego, California, USA; 3 Anlitiks Inc., 18 Old Colony Dr, 02030, Dover, Massachusetts, USA

**Keywords:** Dementia-related psychosis, antipsychotics, atypical antipsychotics, hallucinations, delusions, safety, tolerability, efficacy, effectiveness

## Abstract

**Objectives:**

To evaluate the comparative efficacy, safety, tolerability, and effectiveness of atypical antipsychotics (AAPs) for the treatment of dementia related psychosis (DRP) in older adults.

**Methods:**

In this systematic literature review (SLR), we qualitatively synthesized evidence on the comparative efficacy (based on neuropsychiatric inventory), tolerability (weight gain), and safety (cerebrovascular adverse events [CVAE], cardiovascular events, mortality, somnolence, extrapyramidal symptoms [EPS]) of AAPs used to treat DRP. We also assessed effectiveness based on all-cause discontinuations and discontinuations due to lack of efficacy or adverse events (AE). Published articles from through March 2021 from PubMed, EMBASE, PsycINFO, and Cochrane databases evaluated. We included double-blind, active-comparator/placebo-controlled randomized trials, open-label trials, and observational studies.

**Results:**

This qualitative synthesis included 51 eligible studies with sample size of 13,334 and mean age of 79.36 years. Risperidone, olanzapine, quetiapine, and aripiprazole demonstrated numerically small improvement in psychotic symptoms among patients with DRP. Somnolence was the most reported AE for all the AAPs, with weight gain and tardive dyskinesia more common with olanzapine and risperidone, respectively. These AAPs are associated with falls, EPS, cognitive declines, CVAE, and mortality. Aripiprazole and olanzapine had lower odds of discontinuation due to lack of efficacy, with olanzapine having greater discontinuation odds due to AEs.

**Conclusion:**

This SLR demonstrated that AAPs used off-label to treat DRP are associated with small numerical symptom improvement but with a high risk of AEs, including cognitive decline and potentially higher mortality. These results underscore the need for new treatments with a favorable benefit-risk profile for treating DRP.

## Abbreviations

NPS: Neuropsychiatric symptoms
DRP: dementia-related psychosis
AP: antipsychotics
SLRs: systematic literature reviews
FDA: Food and Drug Administration
AAPs: atypical antipsychotics
EPS: extrapyramidal symptoms
APA: American Psychiatric Association
NPI: neuropsychiatric inventory
NPI-NH: neuropsychiatry index-nursing home
BPRS: Brief Psychiatric Rating Scale
CVAE: cerebrovascular events
AD: Alzheimer's disease
VaD: vascular dementia
DLB: dementia with Lewy bodies
MD: mixed dementia
PD: Parkinson's disease
NH: nursing home
LTC: long-term care
CGI: clinical global improvement
CATIE-AD: clinical antipsychotic trials of intervention effectiveness- Alzheimer's disease
CGI-S: clinical global impression scale- severity
CGI-C: clinical global impression scale- change
PANSS: Positive and Negative Syndrome Scale
SAS: Simpson-Angus Scale
TEAEs: treatment-emergent adverse events
MMSE: Mini-Mental State Examination
BEHAVE-AD: Behavioral Symptoms in Alzheimer's Disease
SUCRA: surface under cumulative ranking curve

## Background

**W**orldwide, an estimated 50 million people currently live with dementia, which results in progressive loss of cognitive function severe enough to cause a decline in the patient's ability to perform activities of daily living (1). It is estimated that 7.5M individuals have dementia of different types in the United States, and this number is expected to double by 2030, given the increase in the elderly population and rising life expectancy (2). While cognitive decline is the hallmark of dementia, neuropsychiatric symptoms (NPS) are common and can dominate its clinical presentation (3). NPS in patients with dementia includes dementia-related psychosis (DRP) that manifests as delusions (false fixed beliefs) or hallucinations (seeing or hearing things that others do not see or hear), and other behavioral symptoms such as agitation, aggression, depression, apathy, elation, anxiety, disinhibition, irritability, and aberrant motor behavior (4). Notably, about 2.4M people in the U.S. are estimated to have DRP (5). Data on patients with Alzheimer's disease (AD) dementia suggest that 94% of patients experience delusions while 70% experience hallucinations (6, 7). DRP prevalence is also anticipated to grow significantly with the increasing rates of dementia, exerting significant distress to individuals and their families and potentially imposing an enormous economic burden to the society (8). Thus, interventions aimed at treating DRP could tremendously improve the health outcomes of patients, their families, and caregivers (3).

At the time of this analysis, there was no Food and Drug Administration (FDA) approved treatment for DRP. However, antipsychotics (APs), both typical antipsychotics (first-generation) and atypical antipsychotics [AAPs] (second-generation) are used offlabel to treat hallucinations and delusions associated with DRP. Available evidence on the comparative efficacy of currently used AAPs versus placebo suggests that AAPs only offer a numerically small improvement in psychotic symptoms among patients with DRP (3). On the other hand, they are known to be associated with significant safety risks related to treatment-emergent cerebrovascular adverse events (CVAE) such as stroke, and a higher risk of mortality (9, 10). In recognition of the unfavorable benefit-risk profile of current off-label AAPs, they all carry an FDA boxed warning on the increased risk of mortality among the elderly with dementia (9). Furthermore, years of research on these medications suggest that each of them report a unique side effect profile that ranges from extrapyramidal symptoms (EPS) (more associated with typical APs) to weight gain, hyperlipidemia, impaired glucose metabolism including potential insulin resistance, diabetes, tardive dyskinesia, sedation, hyperprolactinemia, orthostatic hypotension, sexual dysfunction, increased rate of fractures and cognitive deterioration, among others (11, 12, 13). Considering this, the American Psychiatric Association (APA) guidelines recommend the short-term use of pharmacological interventions only after nonpharmacological interventions such as cognitive, behavioral, and environmental therapies have been attempted first to treat NPS such as DRP (14). Additionally, they also provide instructions for gradual dose reduction or taper.

This study focused on assessing the outcomes of the off-label use of AAPs in treating DRP. Previously published SLRs and network meta-analyses (NMA) assessed AAPs' relative benefits and safety on a broader NPS population from randomized controlled trials (RCTs) which assessed fewer outcomes (15, 16). As they evaluated the overall NPS of dementia, previous studies assessed total neuropsychiatric inventory (NPI) or neuropsychiatry inventory-nursing home (NPI-NH) score as opposed to their psychosis subscale which would be more informative in assessing DRP. The SLRs demonstrated that the trade-offs between the benefits of treating NPS did not adequately offset the risks associated with AAPs. While Yunusa et al. 2019 (15) found significant benefits for total NPI score, and Brief Psychiatric Rating Scale (BPRS) with some AAPs compared to placebo; there were a greater risk of adverse outcomes of CVAE. A recently published SLR and NMA by Watt et al. (16) suggest that, in subgroups of persons with dementia, AAPs are associated with greater harm (i.e., falls, fractures, and CVAE) than antidepressants and anticonvulsants (medications used in place of AAPs for treating NPS of dementia). Other agent (anticonvulsants, dextromethorphan-quinidine) combinations were also associated with an adverse safety profile compared to placebo. However, no single comprehensive SLR has been conducted to comparatively assess efficacy, tolerability, safety, and, most of all, AAPs' effectiveness in treating symptoms of DRP. Furthermore, it is crucial to consider that additional clinical trials have been published since the most recent SLR (17). This SLR aimed to comprehensively compare the efficacy, effectiveness, safety, and tolerability of different AAPs used to treat DRP by including open label and non-double blind studies along with RCTs to assess a broader gamut of outcomes to reflect real-world data and address critical knowledge gaps and provide the most recent review to date.

## Methods

**Figure 1:**
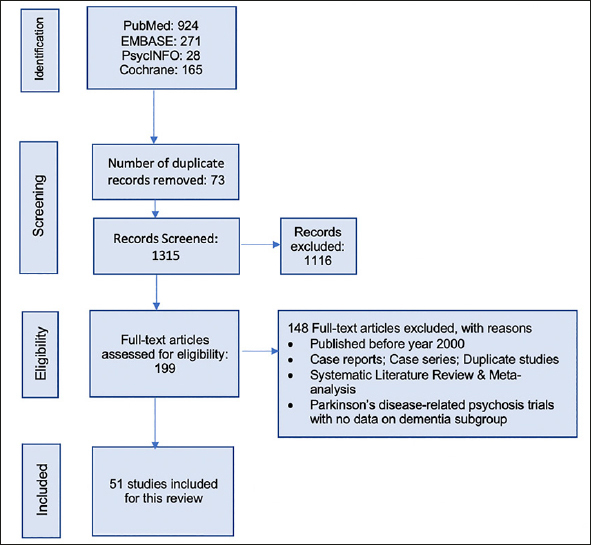
PRISMA Study Selection Flowchart

This systematic literature review followed the preferred reporting items for systematic reviews and meta-analyses (PRISMA) 2020 guidelines (18). The study selection process is illustrated in Figure 1. A protocol for this SLR was developed internally by the study team before starting the review, and it guided the conduct of the study.

### Eligibility Criteria

The Patients/Population, Intervention, Comparator, Outcome, and Study design (PICOS) framework, was used as an eligibility criterion to search, select, and review relevant studies. Included participants from the studies (age ≥ 40, those living in the community or nursing home [NH]) had to have dementia of the following type: AD dementia, frontotemporal dementia, vascular dementia (VaD), dementia with Lewy bodies (DLB) and Parkinson's disease (PD) dementia. The interventions included were both typical and atypical antipsychotics with or without multiple comparator groups and outcomes related to efficacy, tolerability, safety, and effectiveness. Finally, we excluded studies other than double-blind, active-comparator or placebo-controlled RCTs, open-label trials, or observational studies. Efficacy was assessed as improvement in DRP symptoms related to psychosis (i.e., hallucinations and delusions) and the NPI-psychosis subscale, NPI-NH psychosis subscale, BPRS psychosis factor subscale, and BEHAVE-AD (Behavioral Symptoms in Alzheimer's Disease) psychosis subscale were used to measure improvement in psychotic symptoms in patients. The psychosis subscale is a combination of the delusion and hallucination subscales. Additionally, measures of adverse effects were assessed as tolerability (i.e., weight gain due to AAPs) and safety outcomes (i.e., somnolence, EPS including tardive dyskinesia, cognition, CVAE, falls, and mortality, among others). Effectiveness was summarized from all-cause discontinuations and discontinuations due to lack of efficacy or safety.

### Data Sources and Literature Search Strategy

The literature search was conducted in MEDLINE/PubMed (Appendix. 1), PsycINFO, EMBASE, and Cochrane Central Register of Controlled Trials from January 2000 to March 2021. The search was limited to articles published in English. Databases were searched using predefined search terms to identify published studies evaluating the safety and effectiveness of antipsychotics used to treat DRP of any type (PD dementia, VaD, DLB, AD dementia, and frontotemporal dementia). Search strategies were developed using medical subject headings (MeSH) terms (PubMed and Cochrane library), Emtree terms (EMBASE and PsycINFO), and text words related to antipsychotic treatment in DRP.

Key search terms to define the patient population included dementia, NPS, DRP of any type including psychosis related to PD dementia, VaD, DLB, AD dementia and frontotemporal dementia, hallucinations, delusions, agitation, and aggression. Search terms for interventions (medications) included typical antipsychotics, haloperidol, acetophenazine, carbamazepine, chlorpromazine, chlorprothixene, fluphenazine, loxapine, mesoridazine, molindone, perphenazine, prochlorperazine, promazine, thioridazine, thiothixene, trifluoperazine, atypical antipsychotics, aripiprazole, clozapine, ziprasidone, risperidone, quetiapine, olanzapine, pimavanserin, asenapine, brexpiprazole, cariprazine, paliperidone, and lurasidone.

### Search, Study selection, Data Extraction

Two researchers independently searched the indexed electronic databases through the Anlitiks SLR platform, a proprietary internal search engine within Anlitiks that allows integrated searches of selected index databases such as Medline/PubMed and allows the searches for the index databases such as Cochrane review separately, as applicable. Articles were also independently screened against predefined eligibility criteria in two phases, title/abstract screening (Phase 1) and full-text screening (Phase 2). References of all eligible articles were searched to identify the possibility of any missing articles. Subsequently, we extracted data from eligible articles that passed Phase 2 screening using an apriori standardized data extraction form in Microsoft Excel. A third reviewer resolved any disagreement in the information extracted from the articles by both researchers. Data were extracted from both the secondary analysis and original trials. In case of missing information, authors were contacted where necessary. Outcomes related to efficacy, safety, tolerability, and effectiveness were reviewed. Effectiveness outcome was assessed as AP withdrawal or discontinuations, time to AP discontinuations, AP switches or augmentation, as well as relapses of NPS. Also, the availability of data on other effectiveness measures such as hospitalizations, ER visits, and other health resource use was evaluated. Lastly, outcomes like measures of cardiometabolic disturbances (e.g., hyperglycemia, dyslipidemia), sedation, somnolence, and cardiovascular events (e.g., heart attacks), CVAE (e.g., stroke), EPS including tardive dyskinesia, falls and fractures, urinary incontinence, urinary tract infection, as well as measures of cognitive decline, were assessed. In cases where outcomes were reported as a composite measure (e.g., CVAE), we also tried to extract data for the individual outcomes (i.e., stroke, transient ischemic attack, etc.) constituting the composite measure, if available in the published articles. Outcome data were extracted regardless of the reporting format, i.e., either as a dichotomous, categorical, or continuous variable.

### Risk of bias and Study Quality Assessment

**Figure 2:**
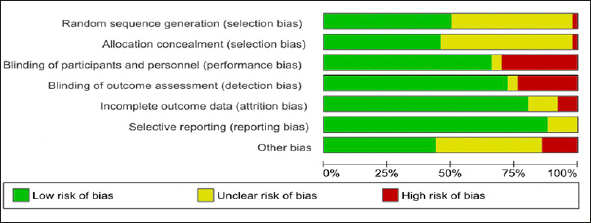
PRISMA Study Selection Flowchart

The Cochrane Risk of Bias tool (19) was used to assess the risk of bias in 49 original trials using seven domains (namely sequence generation, allocation concealment, blinding of participants and personnel, blinding of outcome assessment, incomplete outcome data, selective outcome reporting and ‘other sources of bias's). Accordingly, the risk of bias was categorized on a three-level categorical scale viz. - ‘Low risk' of bias; ‘High risk' of bias, or ‘Unclear risk' of bias. The summary of the risk of bias assessment is shown in Figure 2, and the study-level risk of bias is shown in a tabular form in Appendix 2. The observational studies were assessed using the Newcastle-Ottawa scale for the assessment of the quality of observational studies (20) as shown in Appendix 3.

The figure depicts pooled study quality assessment of all the RCT included in the study.

## Results

### Study Selection

The initial search yielded a total of 1,388 citations, and after removing the duplicates, 1,315 titles and abstracts were screened for eligibility against pre-defined inclusion/exclusion criteria. A total of 199 articles were eligible for full-text review. Overall, 51 studies 17, 21–77) published between January 2000-March 2021 were included in the qualitative synthesis that met the inclusion/exclusion criteria.

### Study Characteristics

Figure 1 illustrates the search yield and study attrition for the selection of eligible studies. Both checklist (Appendix 4,5) (18) and flow diagram from the PRISMA ensured transparency in selecting articles, and the quality standards were followed. Of the total included 51 original studies, 39 were randomized trials, 10 were open-label trials, and 2 were observational studies. Information from 8 post-hoc analyses of the randomized trials were also extracted. Among the identified studies, 21 were conducted in institutionalized settings such as the NH or long-term care (LTC) facilities, while the rest were conducted among community-dwelling, or outpatient settings. Based on assessment of study quality, it was found that 10% of the studies included in the SLR were of low quality.

From the included studies ranging from 4 to 52 weeks, there were a total of 13,334 patients (sample size ranged from 10 to 4499) with mean age of 79.36 years. Most patients in the included studies were diagnosed with AD dementia, with some studies on VaD, DLB, PD-related dementia, or mixed dementia (MD). There were two studies that had PD dementia-related psychosis as their primary inclusion criteria. Of the 42 parallel-group studies, 21 were placebo-controlled studies, and 21 had active-controls. Overall, there were 30 trials of risperidone, 14 of quetiapine, 10 of olanzapine, 3 of aripiprazole. There was one trial each for ziprasidone, tiapride, and brexpiprazole. The summary of all included study characteristics is described in Table 1, and the Table 2 depicts the outcome measures assessed for individual AAPs.

**Table 1:** Summary of individual study characteristics

Author, Year (Study Name/Trial No.)	Study design	Study setting	Study duration (in weeks)	Study population	Sample size	Age (Mean [±SD] or Median [IQR])	Intervention (Dose)	Comparator (Dose)	Findings
Ballard, 2005 (22)	RCT (double blind)	NH	26	AD dementia, dementia with agitation	93	83.8 (7.7)	Quetiapine (25–50 mg/ 12 hours) Rivastigmine (3–6 mg/ 12 hours)	Placebo	NR
Ballard, 2008 (21) (DART-AD Trial/ ISRCTN33368770)	RCT (double blind)	NH	26	AD dementia	165	84.8 (7.0)	Continue neuroleptic treatment for 12 months	Switch to placebo	NR
Barak, 2011 (23) (NCT 01119638)	RCT (double blind)	Inpatients	6	AD dementia with agitation and psychosis	40	78	Escitalopram (10 mg/day)	Risperidone (1 mg/day)	NR
Brodaty, 2003 (25); Brodaty, 2005 (24); Rabinowitz, 2007 (51)	RCT (double blind)	NH	12	AD dementia, VaD or mixed dementia with agitation, aggression, and psychosis	345	83.2 (0.51)	Risperidone (0.25–2mg/ day)	Placebo	BEHAVE-AD psychosis subscale risperidone vs. placebo, mean change (−5.2 vs. −3.3; p=0.04)
Chan, 2001 (26)	RCT (double blind)	Inpatients, Outpatients	12	AD dementia, VaD or a combination of both with behavioral disturbance (unspecified)	58	80.5 (8.2)	Haloperidol (0.5–2mg/day)	Risperidone (0.5–2mg/ day)	BEHAVE-AD psychosis subscale score for risperidone Baseline vs. Endpoint (1.4±2.0 vs. 0.3±0.9)
De Deyn, 2004 (31)	RCT (double blind)	NH	10	AD dementia and delusions or hallucinations	652	76.6 (10.4)	Olanzapine (l–7.5mg/day)	Placebo	Mortality reported within groups: olanzapine 1.0=4; olanzapine 2.5=3; olanzapine 5.0=5; olanzapine 7.5=3; placebo=2
De Deyn, 200530	RCT (double blind)	NH	10	AD dementia with psychosis	208	81.5	Aripiprazole (5–15 mg/d)	Placebo	AEs reported for aripiprazole vs. placebo: Somnolence (8% vs. 1%); UTI (8% vs. 12%); hypertension (4% vs. 5%)
De Deyn, 2011 (32)	RCT (double blind)	NH	6	AD dementia with psychosis and / or agitation.	100	80.1 (6.5)	Quetiapine XR (50–300mg/ day)	Quetiapine IR (25–300mg/day)	AEs reported for quetiapine XR vs. quetiapine IR: Somnolence (14.7% vs. 18.8%); UTI (8.8% vs. 3.1%); Dizziness (1.5% vs. 9.4%); Hemorrhage (0% vs. 9.4%)
Deberdt, 2005 (33)	RCT (double blind)	Outpatient, NH	10	AD dementia, VaD, or mixed dementia with psychosis	495	79.8 (7.2)	Olanzapine (2.5–10mg/day) Risperidone (0.5–2mg/day)	Placebo	AEs reported for olanzapine vs. risperidone vs. placebo: Somnolence (23.2% vs. 18.9% vs. 8.5%)
Devanand, 2012 (34) (ADAD trial)	Randomized, Open label study	Outpatient, NH	16	AD dementia with agitation, aggression, and psychosis	180	80.7 (7.9)	Risperidone (0.25–3mg/ day)	Placebo	Relapse rate for risperidone vs placebo: Until week 16 (33% vs. 60%; P=0.004; HR with placebo, 1.94). Week 17–32 (48% vs. 15%; P = 0.02; HR, 4.88).
Ellingrod, 2002 (35)	Single blind, multicenter observational study	NH	8	AD, multi-infarct dementia, or mixed syndrome and psychosis	19	84.83 (2.07)	Risperidone (0.25–3mg/ day)	Olanzapine (2.5/15mg/ day)	NR
Freund-Levi, 2014 (36)	Randomized, Open label study	Inpatient	12	AD dementia, VaD, frontal lobe dementia, PD dementia with BPSD (unspecified)	100	78.7 (7.5)	Galantamine (4–12 mg/ day)	Risperidone (0.25–1.5 mg/day)	AEs reported for galantamine vs. risperidone: Cardiovascular disorder (12% vs. 20%); Psychiatric disorders (2% vs. 0%); Central and peripheral nervous disorders (12% vs. 10%)
Fujikawa, 2004 (37)	Open label study	Inpatient and outpatient	8	AD dementia with behavioral disturbances	16	81.5 (6.2)	Quetiapine (25–200mg/ day)	N/A	BEHAVE-AD subscale scores for quetiapine Baseline vs. Endpoint; delusion (4.5±3.91 vs. 1.44±1.66); hallucination (0.75±1.03 vs. 0.13±0.33)
Gareri, 200438	RCT (double blind)	NR	8	AD dementia, VaD or a combination of both	60	82.5 (9.3)	Risperidone (1–2 mg/day) Olanzapine (5–10mg/day)	Promazine (50–100mg/ day)	NR
Grossberg (study 1), 2019 (17) (NCT01862640)	RCT (double blind)	NH, Outpatient	12	AD dementia with agitation	433	73.8 (8.7)	Brexpiprazole (l–2mg/day)	Placebo	NR
Holmes, 2007 (39)	RCT (double blind)	Outpatient	6	AD dementia with agitation	28	86.15 (5.8)	Risperidone (lmg/day)	Rivastigmine (3–6mg/day)	Percent of AEs reported in risperidone vs. rivastigmine (33% vs. 60%)
Jeste, 2000 (40)	Open label study	Inpatient	12	AD dementia, VaD, mixed dementia	330	82.5 (7.5)	Risperidone (0.5–2mg/day)	Placebo	NR
Katz, 1999 (76); Rabinowitz, 2007 (51)	RCT (double blind)	NH	12	AD dementia, VaD or Mixed dementia	625	82.7 (7.7)	Risperidone (0.5–2mg/day)	Placebo	The rate of fall were: placebo, 22.3%; risperidone 0.5mg/day, 18.0%; risperidone 1mg/day, 12.7%; and risperidone 2mg/ day, 27.3%.
Kinon, 2003 (77)	Open label study	NH	8	AD dementia, VaD, unspecified dementia	84	83.03 (6.8)	Olanzapine (2.5–10mg/day)	antipsychotic naïve, previously taking conventional antipsychotics, previously taking Risperidone	NR
Kurlan, 2007 (41)	RCT (double blind)	NH, outpatient	10	AD dementia with parkinsonian features, DLB, PD with dementia	40	74.1 (6.1)	Quetiapine (25–300mg/day)	Placebo	AEs reported for quetiapine vs. placebo: Cardiac disorders (7.7% vs. 0%); Nervous system disorders (45% vs. 15%); Vascular disorders (15% vs. 0%)
Kurz, 2005 (42)	Open label study	NR	8	Dementia patients with chronic aggression and psychosis	4499	81 (7)	Risperidone (l–2mg/day)	N/A	AEs reported for risperidone: Death (1.4%); EPS (0.89%); Sedation (0.53%); Cardiovascular (0.38%); Falls (0.31%); CVAE (0.36%)
Laks, 2001 (43)	Open label study	Outpatient settings	16	AD dementia and VaD with agitation	26	76.35 (8.63)	Risperidone (0.25–3mg/day)	None	NR
Lim, 2006 (44)	Randomized, Open label study	Inpatient, Outpatient	8	AD dementia	28	74.2 (5.7)	Amisulpride (50–400mg/day)	Risperidone (0.5–4mg/day)	AEs reported for amisulpride vs. risperidone: Somnolence (33.3% vs. 23%); EPS (20% vs. 38.4%); Dizziness (20% vs. 15.3%)
Mintzer, 2006 (45)	RCT (double blind)	NH	8	AD dementia, VaD with psychosis	473	83.4 (7.2)	Risperidone (0.5–1.5mg/days)	Placebo	AEs reported for risperidone vs. placebo: Somnolence (16.2% vs. 4.6%); Fall (11.1% vs. 12.6%); UTI (9.4% vs. 10.1%); CVAE (1.7% vs. 0.4%)
Mintzer, 2007 (46)	RCT (double blind)	NH	10	AD dementia with psychotic symptoms, delusions, or hallucinations	487	82.5	Aripiprazole (2–10mg/day)	Placebo	The rate of EPS reported was: placebo (6%); aripiprazole 2mg (8%); aripiprazole 5mg (7%); aripiprazole 10mg (7%)
Mowla, 2010 (74)	RCT (double blind)	Outpatients	8	AD dementia with behavioral disturbances	48	74.7 (3.0)	Topiramate (25–50mg/day)	Risperidone (0.5–2mg/day)	NR
Mulsant, 2004 (70)	RCT (double blind)	NH	6	AD dementia or VaD or dementia with mixed etiology	86	83.84 (6.9)	Risperidone (0.25–1.5mg/day)	Olanzapine (2.5–10mg/day)	NR
Mullen, 2001 (47)	RCT Open label	Outpatient	16	AD dementia with psychosis, VaD, other	728	45.36	Quetiapine (50–800mg/day)	Risperidone (l–3mg/day)	AEs reported for quetiapine vs. risperidone: Somnolence (31.3% vs. 15.4%); Dizziness (12.7% vs. 6.9%)
Onor, 2006 (48)	Observational study (Cross-sectional)	Outpatient	12	AD dementia, VD, DLB, and mixed dementia	41	76.15 (6.3)	Quetiapine (12.5–50mg/day)	N/A	NR
Paleacu, 2008 (49)	RCT (double blind)	NH	6	AD dementia with agitation, delusion, anxiety, apathy, irritability	40	82.2 (6.4)	Quetiapine (50–300mg/day)	Placebo	NR
Rainer, 2001 (71)	Open label study	Inpatient and outpatient	8	AD dementia, VaD, mixed dementia, or DLB and delusions, hallucinations, agitation/ aggression, irritability, and disinhibition	34	76.4 (10.3)	Risperidone (0.5–2mg/day)	N/A	NR
Rainer, 2007 (52) (IIT50779026)	RCT (Rater-blinded)	Outpatient	8	AD dementia, VaD or mixed with delusions, hallucinations, agitation/ aggression	72	77.74 (5.9)	Quetiapine (50–400mg/day)	Risperidone (0.5–4mg/day)	AEs reported for quetiapine vs. risperidone: Sedation (10.5% vs. 0%); Femur fracture (5.3% vs. 0%); Somnolence (5.3% vs. 0%); Urinary incontinence (5.3% vs. 0%)
Ruths, 2004 (54)	RCT (double blind)	NH	4	Dementia and antipsychotic use	30	83.4 (6.9)	Haloperidol (0.5–1mg/day) Risperidone (0.5–2mg/day) Olanzapine (2.5–5mg/day)	N/A	NR
Ruths, 2008 (75)	RCT (double blind)	NH	4	Dementia patients receiving long term (>3-months) of either risperidone, olanzapine, or haloperidol for BPSD	55	84.1 (7.1)	Cessation of antipsychotic (Intervention); haloperidol, olanzapine, and risperidone	Continued treatment with antipsychotic drugs (Control)	NR
Savaskan, 2006 (72)	Randomized, Open label study	Inpatients	5	AD dementia with at least three of the following: aggression, psychotic symptoms, sleep-wake cycle disturbances, agitation, restlessness or sundowning.	30	82.3 (2.5)	Haloperidol (0.5–4mg/day)	Quetiapine (25–200mg/day)	NR
Scharre, 2002 (55)	Open label study	Outpatients	12	AD dementia with psychosis or aggressive behaviors	10	76.0 (2.0)	Quetiapine (50–150mg/day)	N/A	NR
Street, 2000 (7;) Cummings, 2002 (29); Mintzer, 2001 (69); Clark WS, 2001 (27)	RCT (double blind)	NH	6	AD dementia with agitation, delusions, or hallucinations	206	82.8 (6.5)	Olanzapine (5–15mg/day)	Placebo	The rate of Somnolence reported was: placebo (6.4%); olanzapine 5mg (25%); olanzapine 10mg (26%); olanzapine 15mg (35.8%)
Streim, 2008 (58)	RCT (double blind)	NH	10	AD dementia with psychosis, delusions, hallucinations	256	83.0	Aripiprazole (2–15mg/day)	Placebo	AEs reported for placebo vs. aripiprazole: Somnolence (4% vs. 14%); UTI (11% vs. 14%)
Suh, 2006 (59)	RCT (double blind)	NH	18	AD dementia, VaD or a combination of both	114	80.9 (8.2)	Risperidone (0.5–1.5mg/day)	Haloperidol (0.5–1.5mg/day)	NR
Schneider, 2006 (56); Sultzer, 2008 (60); Vigen, 2011 (65) (CATIE-AD)	RCT (double blind)	Outpatient	36	AD dementia with psychosis, aggression, or agitation	421	77.9 (7.5)	Olanzapine (2.5–5mg/day) Quetiapine (25–50mg/day) Risperidone (0.5mg–1mg/day)	Placebo	24% of olanzapine patients, 16% of quetiapine patients, 18% of risperidone patients, and 5% of placebo patients discontinued their treatment due to intolerability.
Tariot, 2000 (61)	Randomized, Open label study	Outpatient, Inpatient, NH	52	AD dementia with Psychotic symptoms among the patients but primary inclusion is confirmation of PD diagnosis.	184	76.1 (7.3)	Quetiapine (25–800mg/day)	N/A	AEs reported for quetiapine: Somnolence (31%); Dizziness (17%); UTI (11%)
Tariot, 2006 (62)	RCT (double blind)	NH	10	AD dementia, VaD, dementia or mixed with psychosis	284	82.53 (6.81)	Quetiapine (25–600mg/day) Haloperidol (0.5–12mg/day)	Placebo	AEs reported for quetiapine vs. haloperidol vs. placebo: Somnolence (25.3% vs. 36.2% vs. 4.1); UTI (12.1% vs. 10.6% vs. 5.1); Falls (28.6% vs. 28.7% vs. 28.6%); Fractures (2.2% vs. 6.4% vs. 7.1)
Verhey, 2005 (64)	RCT (double blind)	NH, Outpatient	5	AD dementia, VaD dementia, or mixed dementia with agitation	58	82.86 (8.1)	Haloperidol (1–3mg/day)	Olanzapine (2.5–7.5mg/day)	NR
Weiser, 2002 (73)	Randomized, Open label study	NR	20	AD dementia, VaD and both with behavioral disturbances (unspecified)	90	75.6 (7.9)	Rivastigmine (3–12mg/day)	Risperidone (0.5–2mg/day)	Somnolence was measured to be 31% in risperidone group compared to 26% in combination rivastigmine-risperidone and 31% risperidone-rivastigmine group
Yang, 2016 (66)	Randomized open label clinical study	Inpatients	8	AD dementia, VaD dementia or mixed dementia	108	70.2 (6.34)	Tiapride 200mg/day	Risperidone 2mg/day	AEs reported for risperidone vs. tiapride: EPS (5.56 vs. 1.85)
Yoon, 2003 (67)	Open label study	NH	8	AD dementia with behavioral and psychological symptoms	48	75.7 (8.1)	Risperidone (0.25–>1mg/day)	N/A	NR
Zhong, 2007 (68)	RCT (double blind)	NH	10	AD dementia, VaD with agitation	333	83.23 (7.2)	Quetiapine (25–200mg/day)	Placebo	AEs reported for quetiapine 200 mg vs. quetiapine 100 mg vs. placebo: Fall (84.6% vs. 25.8% vs. 26.1%); Somnolence (9.4% vs. 8.1% vs. 2.2%); UTI (7.7% vs. 16.1% vs. 7.6%); EPS (6.8% vs. 4.8% vs. 5.4%)


Abbreviations and full names; RCT- Randomized control trials; NR- No reported findings relevant to the study; NH- Nursing home; DLB - dementia with Lewy bodies; BPSD - behavioral and psychological symptoms of dementia; VaD- Vascular dementia; AD- Alzheimer's disease; PD- Parkinson's Disease; BEHAVE-AD- Behavioral Symptoms in Alzheimer's Disease; AE- Adverse Events; EPS- Extrapyramidal symptoms; UTI- Urinary tract infection; CVAE- Cerebrovascular adverse events; Olz- Olanzapine; N/A- Not applicable

**Table 2:** Table of evaluated outcome measures for AAPs

	Olanzapine	Risperidone	Quetiapine	Aripiprazole	Brexpiprazole	Ziprasidone
Efficacy Measure						
NPI	X	X	X			X
NPI-NH	X			X		
BEHAVE-AD	X	X	X			
Tolerability						
Weight gain	X			X		
Safety						
CVAE	X	X	X	X		
Somnolence	X	X	X	X	X	X
EPS	X	X	X	X		
Falls		X	X			
Injuries				X		
UTI				X		
Cardiovascular events				X		
Mortality		X	X			
CGI-C	X	X	X	X	X	
Effectiveness Measure						
Discontinuation	X	X	X	X	X	X


The table depicts the individual outcomes assessed for every atypical antipsychotic (AAP); Abbreviations: NPI (Neuropsychiatric inventory); NPI-NH (Neuropsychiatric inventory- nursing home subscale); BEHAVE-AD (Behavioral Symptoms in Alzheimer's Disease); CVAE (Cerebrovascular adverse events); EPS (Extrapyramidal symptoms); UTI (Urinary tract infection); CGI-C (Clinical Global Impression - Corrections). The ‘X' represent outcome measures that were studied for corresponding AAPs. The blank cells represent the outcome measures that were not studied for corresponding AAPs.

While the SLR was intended to include outcome measures such as quality of life (QoL), functioning and caregiver burden outcomes, and hospital admissions and emergency department visits, interestingly, little has been reported in the literature about the role of APs and their impact on these outcomes. Of note, the review resulted in a reasonable number of available publications for the six commonly used AAPs (i.e., olanzapine, risperidone, quetiapine, aripiprazole, ziprasidone, and brexpiprazole) and only one AP (i.e., haloperidol). Therefore, this SLR primarily focused on the qualitative assessment of comparative efficacy, tolerability, safety, and effectiveness of AAPs in the treatment of DRP.

### Olanzapine

A total of 10 studies (4 placebo-controlled and 6 active comparator [e.g., risperidone, haloperidol, and promazine] studies) were included in the review (27, 29, 31, 33, 38, 54, 56, 57, 60, 64, 65, 69, 70, 75, 77). Placebo comparison studies suggest that lower doses of olanzapine have a greater effect on improving psychotic symptoms than higher doses (27, 29, 31, 57, 60). RCT of individuals with AD dementia who were retrospectively identified as meeting the DLB criteria (N =29) found that individuals treated with 5 mg olanzapine/day (N =10) showed greater reductions in NPI delusion subscale (−3.8 points) and hallucination (−5.9 points) subscale scores when compared with placebo (N =10) (29). As per De Deyn et al. 2004, in a double-blind study of 652 patients with delusions or hallucinations associated with AD dementia, olanzapine 7.5 mg/day significantly decreased psychotic symptoms (31). While according to Street et al., another randomized placebo comparison study (N =206) of olanzapine (5, 10, or 15mg/day) among AD patients with psychosis and/or agitation/ aggression, it was reported that low-dose olanzapine (5 and 10 mg/d) showed significant improvement on the NPI-NH psychosis total, subscale compared to placebo; on the other hand, improvement with olanzapine (15 mg/ day) was not significantly greater than placebo for this subscale (57). As per the study published by Verhey et al. 2006 (64), olanzapine was found to improve the NPI psychosis subscale score compared to placebo.

Reported AEs associated with olanzapine were high risk of CVAE (33, 60), somnolence (27, 57, 69), weight gain (31, 38), and EPS (56, 57). For example, Street et al. 200057 found that somnolence was significantly more common among all patients receiving olanzapine, but gait disturbance occurred in those receiving 5 or 15 mg/d (57) compared to placebo and 10mg/d of olanzapine. Additionally, the effects of olanzapine on cognitive symptoms were found to be inconsistent. Although Vigen et al. 2011 (65) found that olanzapine and other AAPs (risperidone) were associated with the worsening of cognitive symptoms, Clark et al. 2001 (27) reported that olanzapine was not associated with a decline in cognition compared to placebo. According to Deberdt et al. (33), olanzapine was associated with a significant increase in CVAE and mortality in comparison with placebo.

For effectiveness outcomes, De Deyn et al. 2004 (31) reported that olanzapine had significantly lower rate of discontinuation due to lack of efficacy as compared to placebo. According to the CATIE-AD trial (56, 60, 65), which compared olanzapine, quetiapine, and risperidone to placebo, olanzapine had the highest rate of discontinuation due to AEs.

### Risperidone

A total of 30 studies (8 placebo-controlled, 18 active-comparator [i.e., quetiapine, haloperidol, olanzapine, and rivastigmine, escitalopram, galantamine, promazine, topiramate, amisulpride, citalopram, tiapride], and 4 single-arm) were included in the review (21, 23, 24, 25, 26, 28, 33, 34, 35, 36, 38, 39, 40, 42, 43, 44, 45, 47, 50, 51, 52, 54, 56, 59, 60, 63, 65, 66, 67, 70, 71, 73, 74, 75, 76). Placebo-comparison efficacy studies of risperidone found that in most cases, risperidone was associated with improved psychosis symptoms, compared to placebo (25, 51, 60). In the clinical antipsychotic trials of intervention effectiveness-Alzheimer's disease (CATIE-AD) study, 421 outpatients, were included with AD and psychosis or agitated/ aggressive behavior. Compared to placebo, greater improvements were seen with risperidone in the BPRS psychosis factor subscale (60). In a randomized placebo comparison study of risperidone (n=345), a significant reduction in BEHAVE-AD psychotic symptoms subscale (p=0.004) was seen with risperidone (25). In a secondary analysis of a 12-week, randomized controlled trial of individuals with AD, mean change at endpoint in BEHAVE-AD psychosis subscale was higher in risperidone group compared to placebo (−5.2 vs. −3.3; p=0.039) (24). Another secondary exploratory analysis of data on 479 nursing-home patients with psychosis of AD from three 12-week, double-blind, placebo-controlled clinical trials reported risperidone to be effective on the BEHAVE-AD delusion and hallucination subscales (51). In a multicenter, randomized, double-blind placebo-controlled trial of nursing home residents diagnosed with AD and psychosis (45), both risperidone group and placebo groups showed significant improvements on the BEHAVE-AD psychosis subscale (45). Overall, these studies reported that risperidone is moderately effective in treating various symptoms associated with psychosis. Interestingly, in a study by Deberdt et al. 2005 (33), olanzapine, risperidone, and placebo treatment reported improved NPI-NH psychosis subscale scores, though no significant changes emerged across treatments, including placebo-comparisons (33). Furthermore, significant reductions were found in the NPI hallucination and delusion subscales scores for amisulpride and risperidone according to Lim et al. 2006 (44).

The AE profile of risperidone demonstrated inconsistent results on the various safety outcomes depending on the number and type of measures. Jeste et al. (40) observed that the incidence of persistent tardive dyskinesia with risperidone appeared to be much lower than that seen in elderly patients treated with conventional neuroleptics (40). In a comparative study of risperidone vs. haloperidol, Suh et al. 2006 (59) reported that the risk of antipsychotic-induced Parkinsonism was significantly lower with risperidone (59). Yoon et al. (67) found that risperidone treatment was generally well tolerated, although EPS were noted in a dose-dependent manner (67). On the other hand, Teranishi et al. 2013 (63) found that drug-induced EPS increased significantly in the risperidone group (63) As far as cognitive decline, there were no differences between placebo and the APs, i.e., risperidone, thioridazine, haloperidol, chlorpromazine, and trifluoperazine in the DART-AD trial (21).

In a study of participants with DLB, risperidone experienced higher overall neurologic effects and worsening of neuropsychiatric symptoms (28). Other reported AEs for risperidone included falls,59,63 EPS (26, 38, 44, 56, 59) somnolence (25, 38, 44, 45, 59) and CVAE (42). In the long-term follow-up of the DART-AD trial, Kaplan-Meier estimates of mortality showed a significantly increased risk of mortality for patients among patients randomized to continue antipsychotic treatment on risperidone compared with those randomized to placebo.

In terms of effectiveness measures, results with risperidone were mixed for outcomes such as discontinuations, time to discontinuations, and or treatment augmentation and switches. In a study by Culo et al. 2010 (28), a significantly higher proportion of participants with DLB (68%) discontinued risperidone prematurely than AD (50%) patients on risperidone, and discontinuation rates were comparable in DLB participants with psychosis that were treated with citalopram (71%) or risperidone (65%). According to the CATIE-AD trials (56, 60, 65), it was reported that risperidone had higher rate of all cause discontinuation and discontinuation due to lack of efficacy compared to placebo. In terms of relapse prevention, Devanand et al. 2012 (34) found that risperidone was associated with a lower rate of psychotic relapse than placebo (60% vs. 33%). To our knowledge, apart from the Devanand study, there have been no other studies of psychotic relapse prevention in patients with DRP.

### Quetiapine

A total of 14 studies (6 placebo-controlled, 4 active-comparator [e.g., risperidone, rivastigmine, quetiapine, haloperidol], and 4 single-arm) were reviewed for quetiapine (22, 32, 37, 41, 47, 48, 49, 52, 55, 56, 60, 61, 62, 65, 68, 72). Efficacy studies for quetiapine in improving psychosis, have had mixed reports; while some studies reported favorable effects on symptom improvements, others showed quetiapine to be ineffective in improving psychotic symptoms. For example, Scharre et al. 2002 (55) found that patients on quetiapine showed a significant reduction in NPI-NH delusion subscale scores, after receiving doses of 50 to 150 mg (55). In another 10-week, double-blind, fixed-dose study, elderly institutionalized patients with dementia and agitation randomized to quetiapine 200mg/day, 100mg/day, or placebo, quetiapine 200mg was associated with clinically greater improvements in the NPI-NH psychosis subscale scores.68 Fujikawa et al. 2005 (37) found significant improvements with quetiapine in the BEHAVE-AD subscales of delusions.

However, other studies found that quetiapine did not improve psychosis compared with placebo (41, 62). For example, Tariot et al. 2006 showed that quetiapine, haloperidol, and placebo demonstrated similar levels of improvement in psychotic symptoms (i.e., no difference between placebo), as reported by mean NPI-NH2 (i.e. hallucination and delusion) subscale, in patients with possible AD from baseline to week 10 (62).

The most commonly reported adverse effects for quetiapine were somnolence (61, 62) death (61, 62) CVAE (52), and EPS (37, 49, 55, 56). While Zhong et al. 2007 (68) found that incidence of CVAE, postural hypotension, and falls were similar among quetiapine and placebo groups while mortality was numerically higher in the quetiapine group; however, these rates were not statistically significant (68).

In terms of effectiveness, Tariot et al. 2000 (61) reported that only 89 (48%) patients (n=184) on quetiapine completed treatment through 52 weeks. The main reasons for antipsychotic withdrawal or discontinuations included lack of efficacy (19%), AEs (15%), failure to return for follow-up (13%). Somnolence (31%), dizziness (17%), postural hypotension (15%) and EPS (13%).61 Onor et al. 2007 (48) found that clinically significant orthostatic hypotension (for patients on quetiapine) led to the discontinuation of 5 patients from their observational study (n =41) (48).

### Aripiprazole

There were 3 studies of aripiprazole compared with placebo. Efficacy results for aripiprazole appear inconsistent across studies (Streim et al. 2008; De Deyn et al. 2005; Mintzer et al. 2007) (30, 46, 58). In a randomized, double-blind, placebo-controlled multicenter trial of 487 institutionalized AD patients with psychosis, Mintzer et al. 2007 (46) found that Aripiprazole 10 mg/day showed significantly greater improvements than placebo on the NPI-NH Psychosis Subscale for baseline scores compared to Week 10 scores (−6.87 versus −5.13; p =0.013) and NPI-NH Psychosis response rate (65 versus 50; p =0.019). However, in the study reported by De Deyn et al., (30) compared to placebo aripiprazole showed similar improvements in psychotic symptoms as assessed by NPI psychosis subscale scores but significantly greater improvements from baseline in BPRS psychosis subscale scores at the study endpoint (30). Additionally, Streim et al. 2008 (58) also found conflicting results to that reported by Mintzer (46), with no significant differences in mean change from baseline score on the NPI-NH Psychosis Subscale between aripiprazole and placebo.

As it relates to the safety and tolerability of aripiprazole, Streim et al, reported comparable rates for treatment-emergent adverse events (TEAEs) between aripiprazole and placebo, except for somnolence (aripiprazole, 14%; placebo, 4%) (58). While in the study reported by De Deyn et al. 2005 (30), only mild somnolence was observed. Moreover, the AEs were generally mild to moderate in severity and included (aripiprazole vs. placebo): urinary tract infection (8% vs. 12%), accidental injury (8% vs. 5%), somnolence (8% vs. 1%), and bronchitis (6% vs. 3%) (30). There were no significant differences from placebo in EPS, or clinically significant ECG abnormalities, vital signs, or weight (30). In a study by Mintzer et al. 2007 (46), CVAE was a reported outcome for the aripiprazole-treated population while no patients from the placebo group suffered from the same. Other AEs seen in the aripiprazole group were asthenia, agitation, and EPS (46).

Effectiveness outcomes reported in these studies did not show any clear patterns. In the De Deyn et al. study (30), the number of patients discontinuing due to AEs, lack of efficacy, or withdrawal of consent was similar in the aripiprazole and placebo groups (30). AEs leading to greater than 2% discontinuation in the aripiprazole or placebo group were asthenia (4%, aripiprazole 10mg/dl) and agitation (4%, placebo), respectively (46).

### Brexpiprazole

There was one reported publication of brexpiprazole that reported the results of two separate studies Grossberg et al. 2020 (17) assessed the efficacy, safety, and tolerability of brexpiprazole in patients with agitation in Alzheimer's dementia (AAD) in two 12-week, randomized, double-blind, placebo-controlled, parallel-arm studies. While one study was a fixed-dose study (Study 1: 433 randomized), the second one was a flexible-dose study (Study 2: 270 randomized) of patients with AAD; in a care facility or community-based setting. Since the main focus of our study was psychosis and Grossberg et a., 2020 focused on patients with agitation, only safety outcomes reported in the study were considered for review.

TEAEs among patients receiving brexpiprazole were headache, insomnia, and somnolence. In general, most TEAEs were mild or moderate in severity. The studies found that brexpiprazole 2 mg/day has the potential to be efficacious, safe, and well-tolerated in the treatment of agitation in AD dementia (17). All cause discontinuations reported across both studies did not show numerical differences between brexpiprazole and placebo groups for both studies. However, discontinuation due to adverse events was reported to be higher for brexpiprazole as compared to placebo.

### Ziprasidone

Rocha et al. 2006 (53) evaluated the efficacy and tolerability of ziprasidone in a 7-week open-label trial. For the patients included in the study, the mean NPI delusion subscale score fell significantly from 4.88 to 2.28 i.e., −53% from baseline to day 49 (p < 0.01). However, of the 25 patients who participated, 10 discontinued the study. The main reason for discontinuation was AEs. The most frequent AEs were somnolence, gastrointestinal symptoms, and parkinsonism (39, 53).

## Discussion

Published SLRs of AAP use among dementia patients largely focused on the gamut of NPS (i.e., psychosis - delusions and hallucinations, agitation/aggression, depression, anxiety, and irritability, among others) and selected safety parameters (such as CVAE, stroke, and mortality) from RCTs or observational studies. This qualitative synthesis adds to previously published SLRs in many ways. First, it is the most comprehensive, recent review of APs as a treatment of dementia related psychosis. Second, this SLR is intended to review real-world effects by including publications of RCTs, open-label trials, and observational studies, including singlearm or comparator-arm trials. Third, this is a comparative review of placebo-AP or AP-AP differences in efficacy, safety, tolerability, and effectiveness in treating DRP. The final and most important difference is that this SLR examined effectiveness outcomes such as differences in all-cause discontinuations and discontinuations due to lack of efficacy or discontinuation due to AEs, and time to relapses between the different AAPs.

This SLR showed that off-label therapies such as risperidone, olanzapine, quetiapine, and aripiprazole demonstrated numerically small psychotic symptom improvements among DRP patients; however, only risperidone was reported to have symptom improvements consistently while also showing a significant increase in EPS. This is supported by the fact that although it is not approved by the FDA, for short-term treatment of aggression in AD, risperidone is the only licensed drug in countries like UK, Canada, and Australia if aggression poses a risk or the person has not responded to non-drug approaches (78, 79). Both quetiapine and aripiprazole reported mixed results, and lower dose olanzapine showed greater symptom improvements than higher doses. These results are consistent with the previously published NMA of 17 studies (5373 patients) conducted by Yunusa et al. 2019 (15), comparing different AAPs (risperidone, olanzapine, aripiprazole, and quetiapine) that reported no statistically significant differences between the AAPs in terms of NPI symptoms scores. While no drug-drug differences were found across measures of efficacy and safety among aripiprazole, olanzapine, quetiapine, and risperidone, placebo-drug differences were found for some drugs for specific outcomes. The surface under the cumulative ranking curve estimated relative ranking of treatments from Yunusa's study suggested that aripiprazole might be the most effective and safe AAP and that olanzapine provides the least effect overall; however, these results should be interpreted with caution where point estimates (OR and SMD) show that there is no statistically significant difference between placebo-drug and drug-drug comparisons from the synthesis of the 17 trials.

Interestingly, the current review, consistent with other studies, suggested that olanzapine may potentially exhibit a dose-response relationship in symptom improvements despite demonstrating only slight symptom improvements with pooled doses. Specifically, doses <10mg of olanzapine were significantly better than placebo in terms of neuropsychiatric symptom improvements (NPI), and doses >10mg were not different from placebo according to two studies (31, 57). While a dose range was found to be effective, no single effective dose was reported in these primary studies. It is not clear why higher doses of olanzapine had similar effects to placebo; it is plausible that the higher rates of AEs reported for olanzapine may have tempered with the effectiveness of olanzapine in psychotic symptom improvements. An NMA along with a meta-regression of these outcomes by dose level differences may be contemplated in the future to test the hypothesis.

While published SLRs and NMAs by Yunusa (15) and Watt (16) suggest that APs may be associated with a significant risk of strokes, falls, fractures, CVAEs, or death, and the umbrella review by Papola et al. (80) suggest an association between the use of APs and fractures, stroke, and cardiac death, the current review of AAP tolerability and safety outcomes included more outcomes such as weight gain, metabolic disturbances (e.g., hyperglycemia, dyslipidemia), sedation, somnolence, and cardiovascular events, CVAE, EPS including tardive dyskinesia, falls and fractures, urinary incontinence, urinary tract infection and cognitive decline. Not surprisingly, our review showed that somnolence was the most reported AE for all the major APs, with weight gain and tardive dyskinesia being more commonly reported for olanzapine and risperidone, respectively. Other AEs reported for all AAPs were EPS, falls, and CVAEs, except for brexpiprazole. Furthermore, studies also show that these APs may be associated with greater cognitive declines (38, 41, 60, 81) and potentially increased mortality (21, 33, 68) in patients with DRP. Although our study was qualitative in nature, Maust et al., reported that, about 27–50 patients need to be treated with AAPs in order for one person to die (9). In alignment with a previous study, this review also suggests that other AEs among patients receiving olanzapine include CVAE and EPS (81). While falls, EPS, and CVAE were also reported for risperidone. In addition to somnolence, commonly reported adverse effects of quetiapine were EPS, dizziness, postural hypotension, and death. AEs associated with aripiprazole are somnolence, urinary tract infection, accidental injury, somnolence, bronchitis, CVAE, akathisia, asthenia, agitation, and EPS. All these qualitative findings suggest the major AAPs that are currently used off-label for treating DRP may have an unfavorable benefit-risk based on multiple outcome measures, including CVAE, and mortality. These findings suggest that a quantitative review through an NMA may be needed.

The current study is an extension of previous reviews by examining additional measures of effectiveness defined as AP withdrawal or discontinuations, time to discontinuations, AP switches or augmentation, as well as psychotic relapses. While our review attempted to review data on other effectiveness measures such as hospitalizations, ER visits, and other health resource use, it was limited by the paucity of data on these outcomes. Available data suggest that compared to placebo, odds of all-cause discontinuations were lower with aripiprazole while olanzapine, quetiapine, risperidone, and brexpiprazole reported no differences. While aripiprazole and olanzapine had lower discontinuation odds due to lack of efficacy, olanzapine had higher discontinuation odds due to lack of safety. Compared to placebo, odds of discontinuation were found to be higher for aripiprazole, olanzapine, risperidone, quetiapine and brexpiprazole. Based on the reported information, it appears that risperidone had a lower likelihood of relapse of NPS than placebo. Studies of olanzapine, quetiapine, brexpiprazole did not report treatment effects based on relapse of psychosis.

For multiple drug comparator studies from the CATIE-AD trial, effectiveness for olanzapine, quetiapine and risperidone was measured through discontinuation or effects of AAP withdrawal. Ruths et al. 2004 (54) reported that for olanzapine and risperidone most patients' behavioral scores remained stable after the withdrawal of APs from nursing home (NH) patients with dementia. However, impairment in the patient's nighttime and daytime activity was reported as sleep problems and restlessness (54). In another double-blind, placebo-controlled trial of 421 outpatients with AD and psychosis or aggression/agitation, Schneider et al. 2006 (56) found no significant differences in time to all-cause discontinuation (i.e., discontinuation for any reason) among olanzapine, quetiapine and risperidone.

As with any research, this SLR has a few limitations. Specifically, the SLR included trials beyond the gold standard for double-blind, randomized trials and thus resulted in 10% of studies of low-quality being included with high risk of bias for blinding of participants and personnel. This could be attributed to the inclusion of non-blinded and open label studies in the SLR. It is anticipated that the small proportion of low-quality studies included in this qualitative review of the SLR are not likely to change the overall conclusions. However, a sensitivity analysis may be considered during quantitative NMA, if any, based on this SLR's findings to evaluate the impact of low-quality studies included in the SLR. It is recommended that the NMA ought to consider issues of heterogeneity, transitivity, and other inherent problems to overcome any intrinsic study-related biases. Notwithstanding these few limitations, this review represents the most comprehensive analysis to-date.

## Conclusions

Consistent with previous findings, the overall evidence from this SLR suggest that currently used AAPs described in this study confer non-significant benefits in treating dementia related hallucinations and delusions. Additionally, they are associated with a high risk of significant AEs, accelerated cognitive decline, and potentially higher mortality among patients with DRP. Furthermore, antipsychotic effectiveness may be poor, given the potentially high rates of all-cause discontinuations and discontinuations due to AEs reported in the studies. Overall, these qualitative findings suggest that these AAPs used as off-label treatments for the vulnerable DRP population may have an unfavorable benefit-risk profile and require quantitative confirmation through an NMA of data derived from this SLR. These results also underscore the potential unmet need for new treatment options with an improved benefit-risk profile for the treatment of DRP.

## Electronic supplementary material

Comparative Efficacy, Safety, Tolerability, and Effectiveness of Antipsychotics in The Treatment of Dementia-Related Psychosis (DRP): A Systematic Literature Review

*Authors' contributions:* IY, NR, VA, KR development of concept and study design; IY, and KR literature search; IY, NR, VA, KR study selection, and interpretation of data; IY, NR, VD, KR preparation of manuscript; All authors critically reviewed and approved the final version of the paper.

*Funding:* The research was funded by Acadia Pharmaceuticals Inc. Employees of Acadia Pharmaceuticals Inc. were a part of the research team for development of concept, study design, study selection and interpretation of the results.

*Data availability statement:* Data sharing is not applicable to this article as no datasets were generated or analyzed during the current study.

*Ethics approval and consent to participate:* Not applicable.

*Consent for publication:* Not applicable.
